# Rapid Progression of Kaposi’s sarcoma complicated with hemophagocytic syndrome in a severely immunosuppressed patient with HIV-infection: a case report

**DOI:** 10.1186/s12981-020-00312-0

**Published:** 2020-09-14

**Authors:** Pingzheng Mo, Liping Deng, Xiaoping Chen, Yong Xiong, Yongxi Zhang

**Affiliations:** Department of Infectious Diseases, Zhongnan Hospital of Wuhan University, Wuhan, 430071 Hubei China

**Keywords:** Kaposi's sarcoma, AIDS, Hemophagocytic syndrome

## Abstract

**Background:**

AIDS-related KS generally involves cutaneous lesions, that slowly progress over months to years. Neither rapidly progressing of KS nor KS complicated with hemophagocytic syndrome (HPS) has rarely been reported.

**Case presentation:**

We report a rare case of rapid progression of Kaposi’s sarcoma complicated with hemophagocytic syndrome in a severely immunosuppressed patient with HIV-infection. The symptoms of this patient were atypical, showing only persistent high fever and rapid progressed to hemophagocytic syndrome. This patient was successfully treated with antiretroviral therapy combined with liposomal doxorubicin.

**Conclusions:**

The condition of the KS patient could deteriorate rapidly over a period of days and even developeded into HPS, which was life-threatening. However, chemotherapy initiated in a timely manner might improve prognosis.

## Backgroud

Kaposi's sarcoma (KS) is a malignant tumor related to acquired immune deficiency syndrome (AIDS) [1]. AIDS-related KS generally involves cutaneous lesions, that slowly progress over months to years. However, it is more likely to involve the viscera or lymph nodes when the patient is severely immunosuppressed [2, 3]. Neither rapidly progressing of KS nor KS complicated with hemophagocytic syndrome (HPS) has only rarely been reported. Recently, a HIV-infected patient with primary manifestation of high fever and rapid progression to shock was diagnosed with KS and HPS. This patient was treated successfully with liposomal doxorubicin. In this report, the clinical presentation, diagnosis and treatment of this case are summarized and reviewed, and relevant literature is reviewed for clinical reference.

## Case presentation

A 32-year-old homosexual male patient with a chief complaint of high fever having lasted for one week was admitted to Zhongnan Hospital of Wuhan University. The fever persisted at 39–41 °C and could be remitted with anon-steroidal antipyretic analgesics, but the temperature rose again after 6-8 h. The fever was not accompanied by chills, cough, abdominal pain, diarrhea, or frequent urination. The patient had received antibiotic treatment for five days in another hospital, but the fever did not resolve and was accompanied by anorexia and weight loss. The positive result of the initial screening for anti-HIV 1 antibodies via enzyme linked immunosorbent assay was positive, which prompted the patient to be transferred to our hospital. There was no history of blood transfusion or intravenous drug abuse. Physical examination showed a body temperature of 38.7 °C, a pulse of 120/min, breathing of 26/min, blood pressure of 96/56 mmHg, anemia and emaciation. The neck was supple, with no superficial lymph node enlargement. No obvious abnormalities were found upon oral cavity and cardiopulmonary examination. The liver and spleen were palpated just under the ribs and found to be soft. There were no visible abnormalities in the chest or abdominal CT scan. Laboratory studies on admission showed white blood cell count of 1.69 × 10^9^/L, hemoglobin of 98 g/L, platelet count of 99 × 10^9^/L, procalcitonin of 1.8 ng/μl and erythrocyte sedimentation rate of 51 mm/L. Liver and renal function were normal except the patient showed low albumin (19 g/L). HIV infection was confirmed by Western blot analysis. The CD4 count was 2 cells/μl and the baseline HIV viral load was 5.0 × 10^5^ copies/ml. Serological detection of (1, 3) β-D-glucan, galactomannan (GM) and interferon gamma release assay (IGRA) were negative. Blood screening for *cytomegalovirus*, *Epstein–Barr virus*, *syphilis*, *hepatitis B virus* and *hepatitis C virus* were negative.

**Fig. 1:**
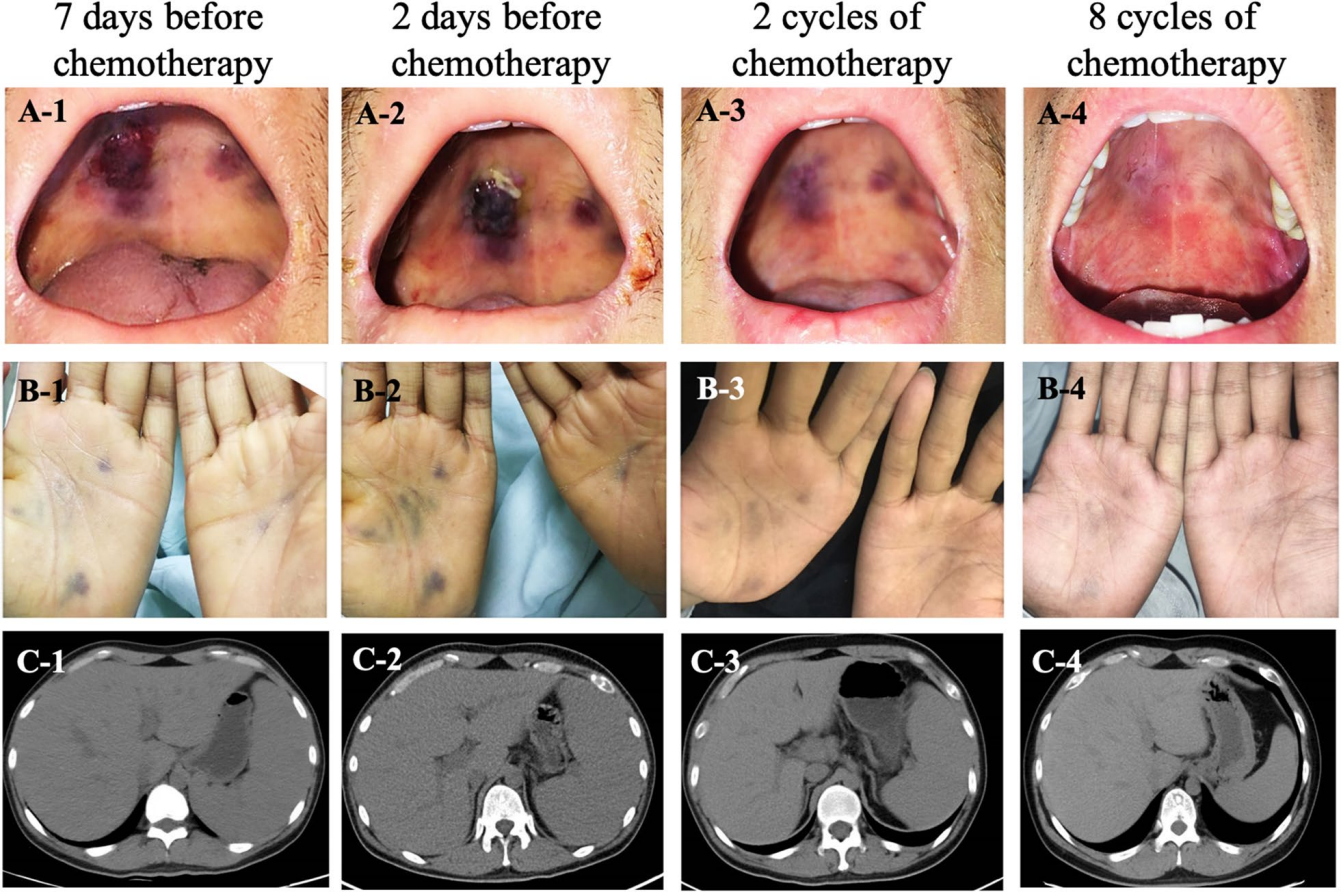
The changes in lesions and spleen before and after chemotherapy. The lesions on oral and palmar surfaces increased both in size and number before chemotherapy (**a-1, a-2, b-1, b-2**), but they faded away after chemotherapy (**a-3, a-4, b-3, b-4**). The abdominal CT scans showed splenomegaly before chemotherapy (**c-1, c-2**), but the size of the spleen decreased to normal (**c-3, c-4**) after chemotherapy

**Fig. 2:**
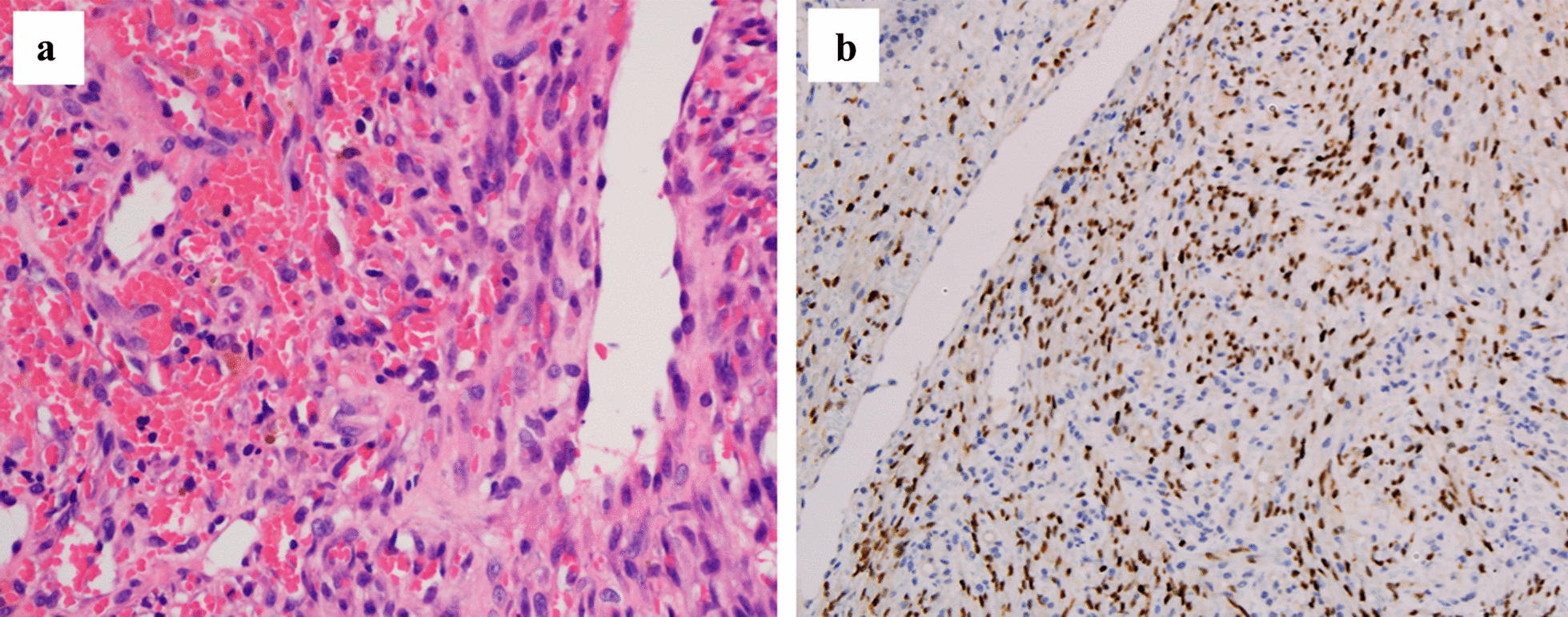
Pathological findings of the nodular lesion on palate. **a** Spindle cells in the dermis grew diffusely with the exudation of a large number of parenchyma vessels and red blood cells (hematoxylin and eosin, 40 ×). **b** Strong and diffuse expression of human herpesvirus-8 was noted (HHV-8 immunostaining, 20 ×)

Diagnosis and treatment: the patient continued to have a fever after admission. Anti-infection treatment started with linezolid (300 mg, Bid), imipenem/cystatin (1.0 g, Q8 h), fluconazole (400 mg, Qd), compound sulfamethoxazole tablets (0.96 g, Qd) and supportive treatment to correct hypotension and hypoproteinemia. On the seventh day after admission, the daily peak body temperature was still at 39–40 °C. He also had poor mental and dietary status. A dark purple linear mucosal lesion approximately 1.5 cm long was found on the left palate with no mass or ulceration. Reexamination of the chest and abdominal CT scan showed bilateral lung infection and splenomegaly. Three sets of blood culture and one set of bone marrow culture were negative. GeneXpert MTB/RIF and GM test in bronchoalveolar lavage fluid (BALF) were both negative. Diagnostic therapy for mycobacteria (rifampicin, isoniazid, moxifloxacin, ethambutol, clarithromycin) were administered for one week. The patient remained hyperthermic, with limited daily function of self-care. On the 17th day after admission, the patient was in a state of shock and his consciousness deteriorated (Glasgow Coma Scale score was 11). He exhibited persistent high fever and dyspnea. An oxygen supply at 10 L/min was administrated in both channels to maintain oxygen saturation at 90% or more. The dark purple mucous membrane lesions on the left palate grew larger, and new dark purple lesions were found on the right palate and bilateral palm surfaces. The spleen was 2 cm below the ribs. Routine blood analysis showed white blood cell count of 0.82 × 10^9^/L, hemoglobin of 57.5 g/L, platelet count of 27 × 10^9^/L. Hemophagocytosis was observed in the second bone marrow aspiration smear. Positron emission tomography–computed tomography (PET-CT) showed enlargement of multiple lymph nodes and splenomegaly (Fig. 1c-2). Supportive treatment was strengthened (subcutaneous injection of the granulate colony-stimulating factors to boost leukocytes, infusion of red blood cells and platelets, administration of dopamine to maintain blood pressure, albumin and plasma to maintain colloid osmotic pressure). Ibuprofen sustained release tablets (300 mg bid) were used to keep the patient’s body temperature below 38.5 °C. On the 19th day after admission, the oral and bilateral palmar lesions had expanded (Fig. 1a-1 and b-1), and four dark purple nodules appeared on his back. Combination antiretroviral therapy (cART, lamivudine /tenofovir disoproxil fumarate/dolutegravir) was started as soon as biopsy of the oral mucosal lesions was performed. On the 23rd day after admission, the nodules on the palate mucosal membrane were further enlarged, and there were multiple purple-black nodules on both palms and lower limbs (Fig. 1a-2 and b-2). The nodules on the back and chest enlarged in size and increased in number. The spleen reached 3 cm below the ribs. The pathological results suggested Kaposi’s sarcoma (Fig. 2), and 40 mg of intravenous doxorubicin liposome was administered immediately. The body temperature returned to normal 3 h after the completion of the first chemotherapy treatment without administering ibuprofen. The next day, WBC, Hb, and PLT values increased, the rash on the palate, back, and limbs did not progress, and the patient’s mental and dietary status improved. This patient was finally diagnosed with AIDS-related KS (T_1_I_1_S_1_ stage) and HPS.

Follow-up: The patient underwent eight cycles of chemotherapy (doxorubicin liposome, 40 mg for 1 day) every 2 weeks, as well as lamivudine/tenofovir disoproxil fumarate/dolutegravir and compound sulfamethoxazole tablets (0.96 g, Qd) every day. The patient’s mental status, diet, sleep and physical strength improved significantly. He gained weight and the rashes on the palate, back, and limbs subsided (Fig. 1a-3, a-4, b-3, and b-4). The chest and abdominal CT scans showed significantly less enlarged lymph nodes in both number and size, as well as a reduction of the size of the spleen (Fig. 1c-3, c-4). Routine blood analysis was normal, and the liver and kidney function, blood sugar, and blood lipids were at normal levels.

## Discussion and conclusions

KS is known as multifocal neoplasm of lymphatic endothelium-derived cells infected with human herpesvirus 8 (HHV-8). Since the widespread use of cART, the incidence of KS has decreased significantly, but it remains one of the most common malignancies in HIV-infected individuals, especially among men who have sex with men [1, 2]. The progression of KS is highly variable. The tumors can remain unchanged for months or years, or they may progress rapidly or disseminate within several weeks. The majority of KS lesions begin on the skin, but they may also appear on oral mucosa, lymph nodes, and internal organs, such as the lungs, liver, spleen, and gastrointestinal tract [3, 4]. The clinical features of KS are lesions that are small papules at first, gradually forming pale red or dark purple plaques and nodules of different sizes. Skin lesions are the main reason patients of KS seek medical advice. However, this patient with AIDS-related KS was admitted to our hospital due to a continuous high fever. No similar lesions were found on the skin, even in other sites. Opportunistic infection, such as mycobacterium, deep fungi and cytomegalovirus, was initially considered to be the most probable cause of high fever. However, no pathogenic microorganisms were detected in the blood, urine, feces, bone marrow and BALF. Multiple imaging examinations, even PET-CT, did not suggest malignancies. Antibiotic, antifungal and anti-mycobacterial treatments were ineffective for the continuous high fever. As the hyperthermia continued for two weeks, the typical skin lesions gradually appeared and spread rapidly through the whole body. Finally, the pathological diagnosis and curative effect showed that KS was the underlying cause of the persistent high fever.

However, the patient’s condition rapidly deteriorated, and the patient developed fever, lymphadenectasis, pancytopenia, coagulopathy, and splenomegaly. Emophagocytosis was observed in a bone marrow aspiration smear and HPS was diagnosed on the third week of fever. HPS is a disorder of the mononuclear phagocytic system caused by dysregulated immune response, eventually resulting in multiple organ failure. Several studies of HPS in HIV-infected individuals with high mortality rates have been published [5, 6]. A retrospective study of HPS in HIV-infected patients revealed that the major etiological triggers in HIV-associated HPS were infection with *Mycobacterium, Cytomegalovirus, Cryptococcus neoformans* and malignancies [7]. HHV-8 is the established causative agent of HIV-associated KS, and it is also recognized as a trigger of HPS in immunosuppressed subjects [3, 5]. In this case, it was likely that HHV-8 infection or KS was related to the occurrence of HPS for reasons given below. First, the immunohistochemical detection of HHV-8 was positive. Second, no other pathogens associated with opportunistic infections were detected. Third, the patient’s body temperature dropped to normal, and the overall condition became stable 3 h after completion of chemotherapy.

cART helps control the progression of KS lesions by inhibiting HIV replication, thereby improving its prognosis [8]. According to the studies, cART increases the five-year survival rate of AIDS-KS patients from 12.1% to 88% [9–11]. For AIDS-related KS patients who have not previously had cART and whose lesions are confined to the skin or mouth, 80% of patients can be effectively relieved after cART administration [3, 12, 13]. In patients with AIDS-related KS who continue to progress after cART treatment or whose lesions involve the internal organs, systemic chemotherapy is required [3, 14, 15]. Doxorubicin liposome is one of the best chemotherapeutic options for KS because of its low toxicity and pronounced anti-tumor effect. Our patient had a disseminated form of KS involving the skin, oral mucosa, pulmonary, bone marrow, and spleen. Based on the staging system developed by the AIDS Clinical Trial Group (ACTG) [14], the staging of AIDS-related KS was T_1_I_1_S_1_. As the condition of this patient rapidly deteriorated and KS came to be highly suspected, cART was administrated first while waiting for pathological confirmation. Doxorubicin liposome systemic chemotherapy was started as soon as the histopathological results became available. The critical condition was rapidly relieved in response to timely treatment.

Treatment modalities of HPS include high-dose glucocorticoids, intravenous immunoglobulin and chemotherapeutic agents. However, studies have shown that glucocorticoids can significantly promote KS cell proliferation by regulating glucocorticoid receptor expression [16]. A number of clinical studies have confirmed that glucocorticoids could increase the incidence, accelerate the progression and increase the mortality of KS in HIV-infected individuals. For these reasons, glucocorticoids are not recommended for AIDS-related KS patients [14, 17, 18]. Liposomal doxorubicin and cART were administrated for this patient of KS complicated with HPS rather than glucocorticoids. Liposomal doxorubicin may improve HPS by suppressing the cytokines production from KS cells. The immunological mechanisms need to be further investigated.

In conclusion, the manifestation of AIDS-related KS could be only high fever at the early stage. These patients with low CD4 cells were easily misdiagnosed with opportunistic infections and treatment might be delayed. Therefore, KS should be suspected whenever there was an unexplained fever, especially accompanied by lesions on the skin or oral mucosa. Furthermore, the condition of the KS patient could deteriorate rapidly over a period of days and even developed into HPS, which was life-threatening. However, chemotherapy initiated in a timely manner might improve prognosis.

## Data Availability

Data sharing is not applicable to this article as no datasets were generated or analyzed during the current study. Additional information is available from the corresponding author on reasonable request.

## Abbreviations

KS: Kaposi’s sarcoma (KS)
AIDS: Acquired immune deficiency syndrome
HPS: Hemophagocytic syndrome
GM: Galactomannan
IGRA: Interferon gamma release assay
BALF: Bronchoalveolar lavage fluid
PET-CT: Positron emission tomography–computed tomography (PET-CT)
cART: Combination antiretroviral therapy
HHV-8: Human herpesvirus 8
